# Total skin electron beam therapy as palliative treatment for cutaneous manifestations of advanced, therapy-refractory cutaneous lymphoma and leukemia

**DOI:** 10.1186/1748-717X-7-118

**Published:** 2012-07-29

**Authors:** Henrik Hauswald, Felix Zwicker, Nathalie Rochet, Matthias Uhl, Frank Hensley, Jürgen Debus, Klaus Herfarth, Marc Bischof

**Affiliations:** 1 Department of Radiation Oncology, University of Heidelberg, INF 400, Heidelberg, 69120, Germany

**Keywords:** TSEBT, Radiotherapy, Irradiation, CTCL, Cutaneous lymphoma, Lymphoma, Leukemia

## Abstract

**Background:**

To retrospectively access the outcome and toxicity of a total skin electron beam therapy (TSEBT) in patients with cutaneous lymphoma (CL) or leukemia.

**Patients and methods:**

Treatment results of 25 patients (median age 63 years; 5 female, 20 male) with cutaneous manifestations of advanced and therapy-refractory CL (n = 21; T-cell lymphomas n = 18, B-cell lymphomas n = 3) stage IIB-IV or leukemia (n = 4; AML n = 2, CLL n = 1, PDC n = 1) treated between 1993 and 2010 were reviewed. All patients were symptomatic. The median total dose was 29Gy, applied in 29 fractions of median 1 Gy each.

**Results:**

The median follow-up was 10 months. Palliation was achieved in 23 patients (92%). A clinical complete response was documented in 13 (52%) and a partial response in 10 patients (40%). The median time to skin progression was 5 months (range 1–18 months) and the actuarial one-year progression-free survival 35%. The median overall survival (OS) after the initiation of TSEBT was 10 months (range 1–46 months) and the actuarial one-year OS 45%. TSEBT related acute adverse events (grade 1 or 2) were observed in all patients during the treatment period. An acute grade 3 epitheliolysis developed in eight patients (32%). Long-term adverse events as a hyperpigmentation of the skin (grade 1 or 2) were documented in 19 patients (76%), and a hypohidrosis in seven patients (28%).

**Conclusion:**

For palliation of symptomatic cutaneous manifestations of advanced cutaneous lymphoma or leukemia, total skin electron beam therapy is an efficient and well tolerated considerable treatment option.

## Introduction

Cutaneous lymphomas (CL) account for approximately 19% of extra-nodal non-Hodgkin’s lymphoma. The predominate form of cutaneous lymphoma in the United States were T-cell lymphoma (CTCL) between 2001 and 2005, which count for approximately 71% of cases, corresponding to an incidence rate (IR) of 7.7/1.000.000 person-years [1]. The subgroup of Mycosis fungoides (MF), which is a T-cell lymphoma primarily of the skin and originating from CD4-positive T-helper cells, accounts for approximately 38% of cases [1]. The WHO-EORTC classification for CL was published 2005 by Willemze et al. and revised in 2010 by Turner et al. [2,3]. The original TNM-classification is found in Tables 1 and 2, but has been revised in 2007 [4]. Extramedullary manifestations of acute myeloid leukaemia are rare, for example Agis et al. reported an incidence of approximately 3% in patients suffering from an acute myeloid leukaemia (AML) [5]. Furthermore, the incidence of granulocytic sarcoma (chloroma) in patients with AML is about 8% [6]. Leukocytic infiltrations to the skin could result in symptomatic plaques and nodules. In CL the clinical appearance includes initially uncharacteristic skin rashes and pruritus, in later stages plaques were found, followed by eventually ulcerating tumors and nodules, as well as erythroderma. Patients might additionally suffer from extensive pruritus, pain and superinfections, so adapted treatments are necessary. Total skin electron beam therapy (TSEBT) using rotational technique was described by Podgorsak et al. and Kumar et al. [7,8]. Furthermore, Funk et al. reported on the technique and setup used in Heidelberg: rotational TSEBT is applied using two 6 MeV-electron beams being reduced to 4 MeV by a Lucite moderator [9]. TSEBT using electron beam energies of 3–4 MeV results in a dose fall-off in the visceral tissue to approximately 10% at 17 mm depth, allowing the application of high therapeutic treatment doses within the skin (approximately 80% dose at 5 mm depth) while avoiding penetration to deeper tissue [7]. For treatment of cutaneous manifestations from leukemia, for example leukemia cutis or chloroma, only a few reports on local radiotherapy or TSEBT do exist and large series or prospective studies are lacking [10-13]. This retrospective single institution analysis is an update of previously published data [9] and was performed to access outcome and toxicity of TSEBT in patients with cutaneous manifestations of advanced, extensively pretreated and therapy-refractory CL or leukemia to help finding ways to improve prognosis, morbidity and mortality.

**Table 1:** classification of cutaneous T-cell lymphoma (CTCL)

**T-classification**	**Description**
T0	Clinically and/or histopathologically suspicious lesions
T1	Limited plaques, papules, or eczematous patches covering <10% of the skin surface
T2	Generalized plaques, papules, or erythematous patches covering 10% or more of the skin surface
T3	Tumors, one or more
T4	Generalized erythroderma
**N-classification**	
N0	No clinically abnormal peripheral lymph nodes; pathology negative for CTCL
N1	Clinically abnormal peripheral lymph nodes; pathology negative for CTCL
N2	No clinically abnormal peripheral lymph nodes; pathology positive for CTCL
N3	Clinically abnormal peripheral lymph nodes; pathology positive for CTCL
**M-classification**	
M0	No visceral organ involvement
M1	Visceral involvement (must have pathology confirmation and organ involved should be specified)

**Table 2:** Staging system of cutaneous T-cell lymphoma (CTCL) (original version)

**Stage**	**T**	**N**	**M**
IA	1	0	0
IB	2	0	0
IIA	1,2	1	0
IIB	3	0,1	0
III	4	0,1	0
IVA	1-4	2,3	0
IVB	1-4	0-3	1

### Patients and methods

#### Patient characteristics

**Figure 1:**
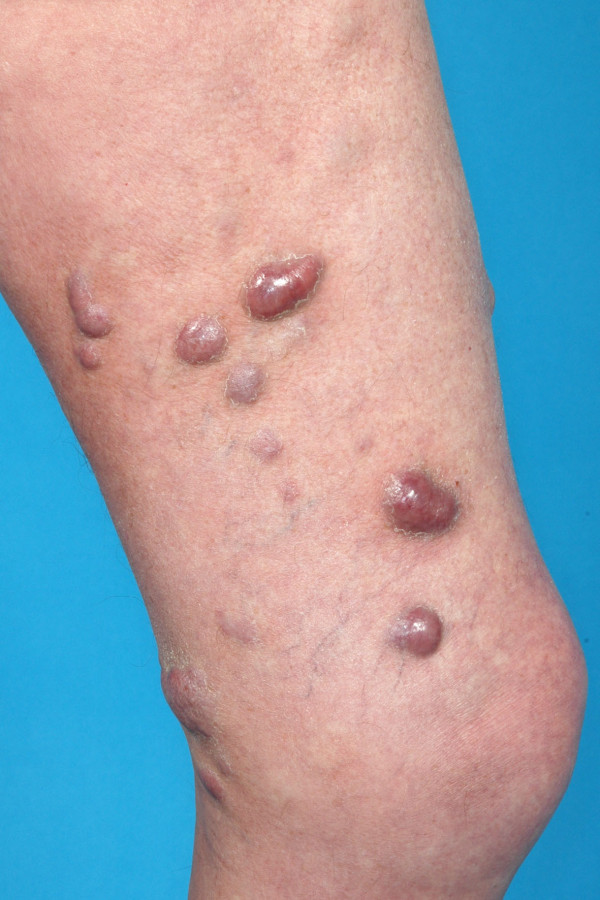
An example of lesions before TSEBT in a patient suffering from stage IIB CL.

**Figure 2:**
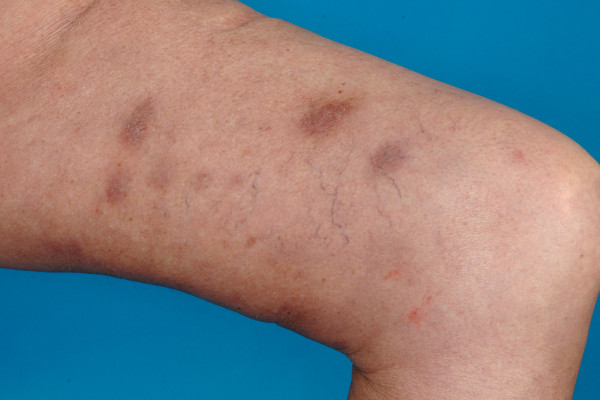
The leasions from image 1 as seen in the first follow-up examination.

Between 1993 and 2010, 25 patients with advanced, pretreated and therapy-refractory CL stage IIB to IV or cutaneous manifestations of leukemia were treated in palliative intention with TSEBT at the Department of Radiation Oncology of the University Hospital Heidelberg. Even though the underlying diseases were partially different, the clinical situations of these patients as well as clinical challenge in palliation were comparable and therefore analyzed together. All patients were refractory to extensive pre-treatment with topical and systemic approaches as chemotherapy, interferon, and/or PUVA-photochemotherapy. All patients were symptomatic with hemorrhages, pruritus, pain and/or ulcerating skin manifestations. The median time interval between initial diagnosis of the underlying disease and initiation of TSEBT was 2 years (range 0.5-8 years). Further characteristics were listed in Table 3. Unfortunately, the retrospective character of this analyses limits further specifications of the underlying diseases. An example of lesions before and in the first follow-up examination in a patient suffering from stage IIB CL were seen in Figures 1 and 2.

**Table 3:** Characteristics of 25 patients with advanced and therapy-refractory CL

**Patient characteristic**	**No. of patients**	**Percentage**
**Demography**		
male	20	80
female	5	20
age:		
median, 63 years		
range, 28-76 years		
**Tumor classification**		
T-cell lymphoma (CTCL)	17	68
B-cell lymphoma (CBCL)	4	16
Acute myeloic leukemia	2	8
(AML)		
Chronic lymphatic leukemia (CLL)	1	4
Plasmacytoid dendritic cell leukemia (PDCL)	1	4
**Tumor stage**		
IIB	4	16
III	1	4
IVA	10	40
IVB	7	28
n. a.	3	12

### Radiotherapy

Radiotherapy was performed as total skin electron beam therapy and the patient standing in an upright position on a rotating base being irradiated by two electron fields with initial energies of 6 MeV that are reduced to 3.8 MeV by a Lucite moderator. Details on treatment setup were published by Funk et al. [9]. It was our intention to perform a conventional TSEBT applying a total dose of 30–35 Gy. The median total dose applied was 29Gy (range 11–35 Gy), median single fraction size was 1 Gy (range 1–1.5 Gy). Additionally, 9 patients received a local boost irradiation using electron beams. During the course of radiotherapy, all patients were treated as inpatients to observe them closely and to avoid a tumor lysis syndrome.

### Statistics

The data was analysed regarding overall survival (OS) and skin-progression-free survival (PFS). Statistical analyses were carried out with SPSS statistical package (SPSS Inc., Chicago, IL, U.S.A.) using log-rank test (Mantel-Cox) and Kaplan-Meier’s estimation. Significance was defined as p-value <0.05. All time estimates began with the initiation of radiation treatment. Documented long-term side effects were classified according to the RTOG/EORTC Late Radiation Morbidity Scoring Scheme (Appendix IV, CTC Version 2.0). Approval of the ethics committee Heidelberg was obtained.

## Results

### Response to treatment, tumor control and survival

**Figure 3:**
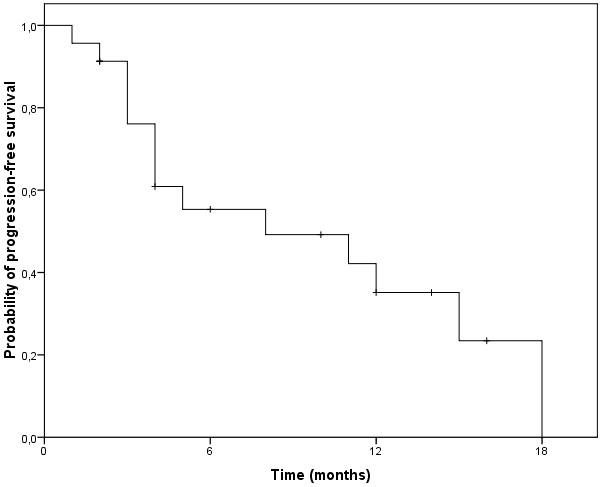
Kaplan-Meier-Estimation of progression-free survival.

**Figure 4:**
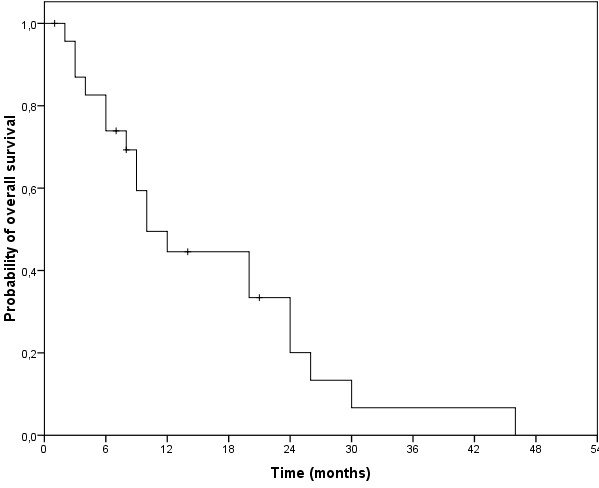
Kaplan-Meier-Estimation of overall survival.

The median follow-up time was 10 months (range 1–46 months). Treatment response regarding palliation with symptom relief, especially of pruritus and regression of cutaneous lesions, was achieved in 23 patients (92%). A clinical complete response was documented in 13 (52%) and a partial response in 10 patients (40%). The median time to skin progression was 5 months (range 1–18 months) and the actuarial six-months and one-year skin-progression-free survival were 55% and 35%, respectively (Figure 3). There were no statistically significant differences (p = 0.076) in PFS between CTCL (mean PFS 8.4 months, 95% CI 5.3-11.5 months), CBCL (mean PFS 3.0 months, 95% CI 1.8-4.1 months) and leukemia (mean PFS 13.5 months, 95% CI 10.6-16.4 months). The median overall survival after the initiation of TSEBT was 10 months (range 1–46 months; Figure 4). The actuarial one-, two- and three-year OS were 45%, 20% and 7%, respectively. In total 8 patients survived at least 20 months after initiation of TSEBT despite advanced and therapy-refractory disease as well as advanced age at time of TSEBT.

### Adverse events

Treatment related acute adverse events as mild to moderate fatigue, mild erythema, dry desquamation, mild edema and alopecia were observed in all patients during the treatment period and shortly after. None of the patients suffered from tumor lysis syndrome. An acute grade 3 epitheliolysis developed in eight patients (31%). Two patients died due to tumor progression and organ failure during the radiation treatment. One of these 2 patients suffered from a pneumonia finally causing fatal renal failure, the other patient developed fatal renal failure due to a blast crisis during TSEBT. Furthermore, following long-term adverse events were documented during follow-up visits: grade 1 or 2 hyperpigmentation of the skin developed in 19 patients (76%), and seven patients (28%) reported a hypohidrosis.

## Discussion

We report the treatment results in 25 patients treated with total skin electron beam therapy between 1993 and 2010 for cutaneous manifestations of advanced, extensively pretreated and therapy-refractory cutaneous lymphoma and leukemia. The overall treatment response with 92% symptom relief, especially of the pruritus and regression of cutaneous lesions, and 52% clinical complete remissions as well as 40% partial remissions demonstrate the efficacy in the setting of advanced and otherwise therapy-refractory disease. In the daily routine, treatment side effects as fatigue and skin reactions seem manageable.

A recent development to reduce the significant treatment-related side effects is to identify a lower possible treatment dose while keeping the effective treatment response. In this setting, Harrison et al. identified 102 patients treated for MF with low-dose (5- < 30Gy) TSEBT between 1958 and 1995. Patients had stage classifications T2-T4. The overall response rates were dose dependent with 90% in patients receiving 5 to <10 Gy (n = 19), 98% in 10 to <20Gy and 97% in 20 to <30Gy and efficacy measures as OS and PFS were comparable [14]. In comparison to our results, these patients were treated with TSEBT median 4 months after initial diagnosis; in our cohort, patients were extensively pretreated and time from initial diagnosis to TSEBT was median 2 years. Besides this, the majority (51 patients) had T2 disease. The PFS was not given for the whole group, but the subgroup with T3 and T4 disease and >/= 30 Gy TSEBT had a median PFS of 2.9 years and 4.6 years, respectively. Kamstrup et al. evaluated prospectively the efficacy of low-dose (10Gy) TSEBT. Ten patients with stage IB-IV MF were treated with 4 fractions of 1 Gy each week to a total dose of 10Gy. Six patients had T2, 2 patients T3 and 2 patients T4 disease, all patients received prior therapies. The median time from initial diagnosis to TSEBT was 1 year. The achieved overall response rate was 90% with a rate of CR as high as 70%. Median PFS was 5.2 months. Treatment was well tolerated with transient alopecia (56%) and ocular irritation (33%) as most common side effects [15]. In comparison to our data, the overall response rates as well as PFS are comparable; however the number of patients with limited disease is relatively high in this study and one would have expected better results in T2 stage.

An actual publication by Lindahl et al. reviewed 35 patients with CTCL treated with TSEBT in Denmark between 2001 and 2008. Almost all patients had previous treatments and 40% of patients had T2 disease. The median follow-up time was 7.6 months and 25 patients were treated with high-dose (30 Gy) TSEBT and 10 patients in a low-dose (4 Gy) protocol. The group of patients with low-dose TSEBT had inadequate treatment response (CR in 10%) compared to higher-doses (26–30 Gy in 1 Gy fractions; CR in 68%). Overall, 28% showed progressive disease, 22.2% in T2 and 35.7% in T3 stage. Median time to progression was 9 months. Side effects related to TSEBT were documented in 88% of patients, including erythema and ulceration in 80%. The most common long-term side effect was alopecia (44%), dry skin (36%), hyperpigmentation (28%), ocular irritation (24%) and temporary loss of finger nails (16%). Furthermore, 2 patients developed basal cell carcinoma and one patient a squamous cell carcinoma. However, in 1 of these 3 patients, PUVA was administered previously, while one other of these 3 patients previously received topical nitrogen mustard. The authors concluded that CTCLs are highly radiosensitive and TSEBT offers significant palliation, but relapse is common and it remains unclear whether prior treatment to TSEBT prolongs the effects of TSEBT [16]. Navi et al. reported on 180 patients treated with TSEBT as a first-line therapy for stage T2 and T3 MF, additionally, many patients received HN2 topically as an adjuvant treatment. All analyzed patients received a total dose of 30 Gy or more, in several patients a second course of TSEBT was necessary. Sixty-three percent achieved a clinical complete remission (CCR), the CCR rates for T2 and T3 stage were 75% and 47%, respectively. Furthermore, freedom from relapse (FFR) was longer in patients with T2 than T3 disease. Median duration of response was 29 months in T2 stage and 9 months in T3 stage. In multivariate analysis T2-stage and fewer prior therapies were associated with improved FFR. The 5-year overall survival was 63%, again, multivariate analysis showed T2-stage and lower count of prior therapies as well as younger age to be prognostic. Regarding side effects, nearly all patients were reported to have mild to moderate dermatitis, alopecia, nail dystrophy and xerosis [17]. In comparison, our results have lower survival rates and earlier tumor progression, which might be explained by the different patient selection. In our department, we normally see patients with advanced, extensively pretreated and therapy-refractory CL (including symptomatic patients with M1 disease), and TSEBT is not commonly used as a first-line therapy. So the patients have had multiple different therapies including chemotherapies and/or PUVA-photochemotherapy and are refractory to those. As Navi and colleagues reported, prior treatments reduced in their multivariate analysis the possibility of response to TSEBT [17]. Possibly native lymphoma cells respond better to TSEBT, which might explain the limited PFS in our analysis in comparison to e. g. Navi and colleagues. Furthermore, the stage of disease as well as extension of the lesions might play a relevant role. The patient group in our analysis included 10 patients in stage IVA disease and 7 patients in stage IVB disease, which were commonly not found in previous reports. An overview of the literature is found in Table 4.

**Table 4:** Overview of literature

**Author**	**Stage**	**Type of radiation treatment**	**Total dose**	**Treatment response**	**PFS**	**OS**
*Lindahl et al.*[16]	T1 5.7%	TSEBT	High-dose (30 Gy) n = 25	High-dose: CR 68%	9 months median	n. a.
	T2 40%		Low-dose (4 Gy) n = 10	Low-dose:CR 10%		
	T3 48.6%					
	T4 5.7%					
*Navi et al.*[17]	T2 57%	TSEBT (+/- HN2)	36 Gy (range 30-40 Gy)	T2: CCR 75%	T2: 8.5 years	T2: 10.9 years
	T3 43%			T3: CCR 47%	T3: 2.9 years	T3: 4.7 years
*Kamstrup et al.*[16]	T2 n = 6	TSEBT	10 Gy	OR 90%	median response duration 5.2 months	n. a.
	T3 n = 2			CR 30%		
	T4 n = 2					
*Chinn et al.*[18]	T2 n = 55	TSEBT (+/- HN2)	Mostly 36 Gy	T2: CR 76%	Freedom from recurrence at 1 year 41%	T2: 10.7 years
	T3 n = 27			T3: CR 44%		T3: 3.6 years
*This Analysis*	IIB n = 4	TSEBT	Median 29 Gy	OR 92%	5 months	10 months
	III n = 1			CCR 52%		
	IVA n = 10					
	IVB n = 7					

**Abbreviations**: *TSEBT* total skin electron beam therapy, *CR* complete remission, *CCR* clinical complete remission, *OR* overall response, *PFS* progression free survival, *OS* overall survival.

In 2009 Neelis and co-workers published results on 18 patients with CBCL treated with palliative low-dose involved-field (IF) radiotherapy (4 Gy in 2 fractions) and 31 patients with MF treated with 4 Gy, later with 8 Gy in 2 fractions. In total 126 lymphoma sites were treated. All patients were previously treated with different approaches, 15 patients have already had TSEBT. In the group of patients with CBCL, the rate of complete remission (CR) was 75% (33/44 lesions). In patients suffering from CTCL and MF, the remission rate after application of 2 x 2 Gy was disappointing (70% failed to achieve a CR), so the treatment dose was increased to 2 x 4 Gy and a CR rate of 92% (60/65 lesions) was achieved. According to the authors, high-grade side effects were not seen. In conclusion, the authors recommended low-dose IF radiotherapy as standard treatment in patients with cutaneous lymphoma, with the option of re-irradiation at progression [19]. From our point of view, this concept of low-dose IF-RT seems to be an option in localized CL, or in the situation of localized recurrence after TSEBT.

Literature on cutaneous manifestations of leukemia, e. g. leukemia cutis is very rare. Pepek and colleagues reported on 2 pediatric patients treated with TSEBT and recommended the consideration of TSEBT for palliation [11]. Furthermore, Rubin et al. concluded in a case report on a pediatric patient treated with TSEBT for leukemia cutis, that TSEBT is feasible and potentially effective [10]. Our experience supports the feasibility of TSEBT in cutaneous manifestations in leukemia, especially in palliative situations.

## Conclusion

For palliation of symptomatic cutaneous manifestations of advanced, PUVA- and chemotherapy-refractory cutaneous lymphoma or leukemia, total skin electron beam therapy is an efficient and well tolerated considerable treatment option.

## Competing interests

The authors declare that they have no competing interests.

## Authors' contributions

HH: analysis and interpretation of data, writing manuscript. FZ: critically revision for important intellectual content, interpretation of data. NR: acquisition and analysis of data. MU: acquisition and analysis of data. FH: critically revision for important intellectual content, interpretation of data. JD: critically revision for important intellectual content, interpretation of data. KH: critically revision for important intellectual content, interpretation of data. MB: substantial contributions to conception and design; critically revision for important intellectual content; final approval for publication. All authors have read and approved the final manuscript.
